# Recent insights into the implications of metabolism in plasmacytoid dendritic cell innate functions: Potential ways to control these functions

**DOI:** 10.12688/f1000research.11332.2

**Published:** 2017-06-22

**Authors:** Philippe Saas, Alexis Varin, Sylvain Perruche, Adam Ceroi

**Affiliations:** 1 EFS Bourgogne Franche-Comté, Université Bourgogne Franche-Comté, Inserm, UMR1098, Besançon, F-25000, France; 2 The Center for Cell Clearance, University of Virginia, Charlottesville, VA, 22903, USA

**Keywords:** plasmacytoid dendritic cells, immunometabolism, cholesterol, fatty acid, LXR, PPAR, type I interferon, glycolysis

## Abstract

There are more and more data concerning the role of cellular metabolism in innate immune cells, such as macrophages or conventional dendritic cells. However, few data are available currently concerning plasmacytoid dendritic cells (PDC), another type of innate immune cells. These cells are the main type I interferon (IFN) producing cells, but they also secrete other pro-inflammatory cytokines (e.g., tumor necrosis factor or interleukin [IL]-6) or immunomodulatory factors (e.g., IL-10 or transforming growth factor-β). Through these functions, PDC participate in antimicrobial responses or maintenance of immune tolerance, and have been implicated in the pathophysiology of several autoimmune diseases, as well as in tumor immune escape mechanisms. Recent data support the idea that the glycolytic pathway (or glycolysis), as well as lipid metabolism (including both cholesterol and fatty acid metabolism) may impact some innate immune functions of PDC or may be involved in these functions after Toll-like receptor (TLR) 7/9 triggering. The kinetics of glycolysis after TLR7/9 triggering may differ between human and murine PDC. In mouse PDC, metabolism changes promoted by TLR7/9 activation may depend on an autocrine/paracrine loop, implicating type I IFN and its receptor IFNAR. This could explain a delayed glycolysis in mouse PDC. Moreover, PDC functions can be modulated by the metabolism of cholesterol and fatty acids. This may occur via the production of lipid ligands that activate nuclear receptors (e.g., liver X receptor [LXR]) in PDC or through limiting intracellular cholesterol pool size (by statin or LXR agonist treatment) in these cells. Finally, lipid-activated nuclear receptors (i.e., LXR or peroxisome proliferator activated receptor) may also directly interact with pro-inflammatory transcription factors, such as NF-κB. Here, we discuss how glycolysis and lipid metabolism may modulate PDC functions and how this may be harnessed in pathological situations where PDC play a detrimental role.

## 1. Introduction

More and more data are available concerning the role of cellular metabolism in innate immune cells, such as macrophages or conventional dendritic cells (cDC) ^1– 3^. However, few data are available currently concerning plasmacytoid dendritic cells (PDC). PDC belong to the family of dendritic cells (DC) and possess specific features that distinguish them from cDC. PDC represent the main type I interferon (IFN) secreting cells and play a critical role in antimicrobial immune responses. The involvement of PDC through IFN-α secretion has also been reported in several autoimmune diseases (see section 2.4). Furthermore, PDC release other pro-inflammatory cytokines, as well as immunoregulatory factors. In these ways, they may exert pro-inflammatory functions or, on the contrary, participate in tolerance mechanisms. In this Review article, based on recent publications ^4– 6^, we will discuss how the innate immune functions of PDC may be modulated by or dependent on the glycolytic pathway (also known as glycolysis) and lipid metabolism, including the metabolism of cholesterol and fatty acids. Before that, we will describe the innate functions of PDC. PDC also have the capacity to present antigens to T cells and to polarize CD4 ^+^ helper T cell responses, as well as those interacting with B cells ^7^. These functions will not be discussed in this article, since no sufficient data are available concerning the impact of metabolism on the capacity of PDC to interact with the adaptive immune system. Although the “immunometabolism” (as defined in Ref# 2) includes six metabolic pathways (glycolysis, the tricarboxylic acid [TCA] cycle, the pentose phosphate pathway, fatty acid oxidation, fatty acid synthesis and amino acid metabolism) that influence immune cell effector functions ^2^, this article will focus on glycolysis and lipid metabolism (extended to the cholesterol metabolism). Concerning amino acid metabolism, PDC innate functions have been shown to be modulated by mammalian target of rapamycin (mTOR) signaling. This central metabolic regulator, mTOR can sense amino acid sufficiency in lysosomes, and promotes mRNA translation and lipid synthesis to support cell growth and proliferation ^2^. Furthermore, mTOR, through its association with regulatory-associated protein of mTOR (RAPTOR), constitutes the mTOR complex 1 (mTORC1), which is connected with other metabolic pathways (see a recent review ^8^ and section 3.1). This will be discussed briefly, since the role of mTOR in innate immune cell functions has been reviewed recently ^3, 8, 9^. Finally, concerning amino acid metabolism, PDC are able to sense amino acid deficiency through their expression of GCN2 (general control nonderepressible 2) serine/threonine kinase. Indeed, the suppression of interleukin (IL)-6 production in PDC by indoleamine 2,3-dioxygenase (IDO) involves GCN2 kinase ^10^ (see section 2.3).

What are the main roles of the immunometabolism in immune cells? First of all, this is a way to provide energy. Cells need energy to execute cellular functions, such as survival, proliferation or cytokine secretion. This energy is provided as adenosine triphosphate (ATP) by several pathways. The first is glycolysis, which involves the conversion of glucose to pyruvate in the cytosol. The second pathway is the TCA cycle (also called the Krebs cycle), which donates electrons to the electron transport chain located in the mitochondria to fuel oxidative phosphorylation or respiration (OXPHOS). This OXPHOS process generates ATP in the mitochondria. Other substrates, such as fatty acids *via* β-oxidation (also called fatty acid oxidation [FAO]), can replenish the TCA cycle to fuel OXPHOS ^11^. In addition to substrates used for energy production and *de novo* biosynthesis, mitochondrial metabolic pathways (such as the TCA, FAO, or OXPHOS) provide substrates for epigenetic modifications of DNA and histones ^12, 13^. This is the case, for instance, of acetyl-CoA for histone acetylation, which is associated with active transcription ^12^. This connects mitochondrial metabolism to epigenetic regulation ^13^. This specific aspect will be briefly discussed in the Conclusions of this article.

## 2. The innate immune functions of plasmacytoid dendritic cells

PDC belong to the DC family and possess specific features that distinguish them from cDC ^14^. These features include: the capacity to rapidly and massively produce type I IFN ( *i.e*., IFN-α/β), the expression of a particular set of pattern-recognition receptors (PRR), leading to the recognition of specific pathogen-associated molecular pattern (PAMP) and damage-associated molecular pattern (DAMP) molecules, as well as a preferential localization in lymphoid organs ^7^.

PDC were firstly identified in human as the major IFN-α producing cells, and thus initially called IPC (IFN-α producing cells) ^15, 16^. After this characterization in human, its murine PDC counterpart was then isolated ^17– 20^. Human PDC are usually identified as CD4 ^+^, CD303 ^+^ (previously known as BDCA-2), CD123 ^high^, and CD11c ^-^, whereas mouse PDC are CD11c ^int^, B220 ^+^, SIGLEC-H ^+^, and CD317 ^+^ (also known as BST2 or PDCA1) ^7^. Despite the difference in phenotypes of human and mouse PDC, PDC from both species exhibit a conserved genetic signature with some common genes (e.g., *tlr7* or *ifr7*) ^21^. Moreover, PDC exhibit specific transcription factors, such as the transcription factor E2-2 (also known as TCF4) or SPIB ^7^. A differentiation/ontogeny process distinct from those of cDC has been reported ^7^.

### 2.1. PDC ontogeny and localization

Development of PDC from hematopoietic stem cells occurs in the bone marrow. After this differentiation step, PDC are released from the bone marrow to the blood for homing to different lymphoid tissues ^22^. Thus, PDC isolated from blood of healthy donors or patients consist in PDC migrating to these tissues ^7^. In steady state, PDC reside mainly in T cell-rich areas in lymph nodes and secondary lymphoid organs ^7^. Localizations of PDC in other lymphoid organs, such as Peyer’s patches of the gut ^23, 24^, and tonsils ^25^, have been reported. PDC residing in non-lymphoid tissue – such as the airways ^26^ and the liver ^27^ – exert a critical role in steady state by regulating mucosal immunity and maintaining tolerance to inhaled or ingested antigens ^28^. Finally, PDC are also present in the thymus during homeostatic conditions, where they play a role in central tolerance ^29– 31^. In contrast, during infections or autoimmune diseases, PDC migrate to inflamed lymph nodes ^15^ or inflamed epithelia ^15, 25, 32^. Moreover, PDC infiltrate several tumors including: melanoma ^33^, head and neck ^34^, breast ^35– 37^ and ovarian ^38– 40^ tumors. The microenvironment in which PDC are present may influence oxygen and nutrient availability, which impacts on metabolic pathways (see section 3).

### 2.2. Type I IFN and pro-inflammatory cytokines secreted by PDC

PDC constitute a DC subset, specialized in antimicrobial immune responses. This occurs mainly through a rapid type I IFN (IFN-α/β) production ^7, 41, 42^. Selective depletion of PDC by genetic approaches supports the critical role of PDC for early IFN-α production after microbial infections ^43– 45^. The type I IFN response is triggered by Toll-like receptor (TLR) signaling after PAMP recognition.

**Figure 1.**
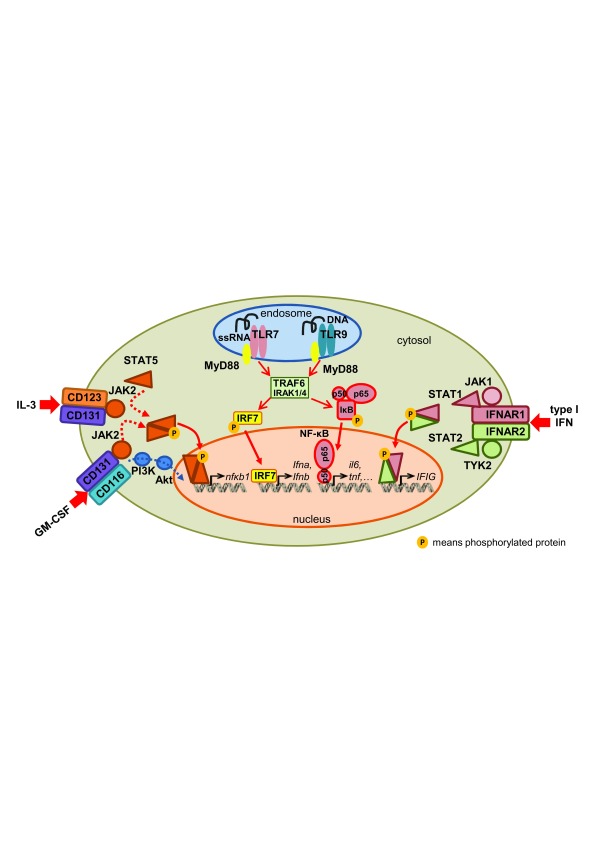
The main signaling pathways in plasmacytoid dendritic cells that promote metabolic changes or are modulated by metabolic pathways. This figure summarizes different signaling pathways described in the literature to promote metabolic changes or to be modulated by immunometabolism in plasmacytoid dendritic cells. This includes: endosomal TLR 7 and TLR9, membrane IL-3 receptor (associating CD131 to CD123), GM-CSF receptor (associating CD131 to CD116), and IFN-α receptor (IFNAR associating IFNAR1 and IFNAR2). Only the main pathways with main effector molecules are depicted. For more details, please refer to the main text. Abbreviations (not defined in the main text): *IFIG*, IFN-I-induced genes; *nfκB1*, NF-κB gene.

Compared to cDC, PDC express a limited number of PRR. TLR 7/9 are expressed by both human and mouse PDC ^7, 46^. These two endoplasmic TLRs allow PDC to recognize cytosine-phosphate guanosine (CpG)-rich unmethylated DNA from bacteria and DNA viruses, ^7, 41, 47^, as well as viral single-stranded RNA (ssRNA), ^41, 48, 49^, respectively. In addition, PDC are able to recognize *via* TLR7/9 mammalian nucleic acids ^50^, in particular when these nucleic acids are complexed or associated with antimicrobial peptides (e.g., LL37) ^51, 52^. Once PDC have sensed pathogens or DAMP through endoplasmic TLR, signaling is mediated *via* MyD88 (myeloid differentiation factor 88), a docking protein for IRAK1⁄4 (IL-1R-associated kinase 1/4), and the ubiquitin ligase TRAF6 (tumor necrosis factor [TNF] receptor-associated factor 6). IFN-regulatory factor 7 (IRF7) is then phosphorylated and translocates into the nucleus to induce *type I IFN* gene and IFN-inducible gene transcription ( Figure 1) ^53^. This is true for human PDC ^46^. Concerning mouse PDC, other intermediates may participate in *type I IFN* mRNA transcription in the TLR-dependent IRF7 signaling pathway. This involves a complex, associating TRAF3, IRAK1, osteopontin, PI3K (phosphatidylinositol 3-kinase) and IKKα (IκB kinase-α) ^46^. A critical role of PI3K has also been reported for type I IFN production by human PDC ^54^. In addition, in TLR7- or TLR9-activated human PDC, TRAF6 can also recruit TAK1 (transforming growth factor [TGF]-β-activating kinase; also known as MAP3K7 for mitogen-activated protein kinase kinase kinase 7) to trigger the synthesis of pro-inflammatory cytokines *via* NF-κB activation ^46^. In mouse PDC, TAK-1/MAP3K7 activates the mitogen-activated protein kinase (MAPK) pathway that upregulates costimulatory molecule expression (e.g., CD40, CD80 or CD86) ^46^. Both human and mouse PDC have been shown to secrete TNF-α ^4– 6, 55, 56^, IL-6 ^4– 6, 55, 56^, IL-8 ^55– 57^ or granulocyte macrophage colony-stimulating factor (GM-CSF) ^55^. This TLR-induced cytokine synthesis is regulated in PDC by the translocation of NF-κB, p38 MAPK and c-Jun N-terminal kinase (JNK) into the nucleus. In human PDC, the association of NF-κB p65 and p50 subunits with IRF5 appears to be the master inducer of *IL-6* mRNA transcription ^46^. Depending on the TLR9 ligand used, the cytokine response can be different. For instance, type A CpG-containing oligonucleotide (CpG-ODN) (CpGA) induces mainly type I IFN production, whereas type B CpG-ODN (CpGB) induces pro-inflammatory cytokine secretion and upregulation of co-stimulatory molecules ^58^.

In addition to virus or pathogen-expressing TLR7/TLR9 ligands or synthetic ligands, PDC can be activated to release cytokines by other stimuli, including DAMP. PDC express RAGE (receptor for advanced glycation end products), a PRR that recognizes high-mobility group box-1 (HMGB1) ^59, 60^, a nuclear DNA-binding protein released from necrotic cells ^61^. PDC can also be activated by neutrophil extracellular traps (NET), released by dying or activated neutrophils. These NET contain DNA fibers, histones, as well as a large amount of LL37 and HMGB1 ^62, 63^. Human PDC can also be activated by CD154 from activated platelets ^64^ or endothelial-derived microvesicles ^6, 56^. Other PRR are present in the cytoplasm of PDC and dedicated to RNA virus recognition. This is the case of retinoic acid-inducible gene (RIG)-I-like receptors, DHX9 or DHX36 ^65^.

### 2.3. Regulatory or immunosuppressive factors expressed by PDC

In addition to type I IFN and pro-inflammatory cytokine production, PDC have regulatory and immunosuppressive functions ^28^. This has been demonstrated by *in vivo* PDC depletion studies ^26, 66^. For instance, PDC exert immunoregulatory functions in the lung, preventing deleterious asthmatic reactions ^26^. PDC have been shown to prevent allo-immune responses in the setting of solid organ transplantation ^67^ or after hematopoietic cell transplantation ^68^. PDC participate also in oral tolerance ^69^. Moreover, PDC may express immunosuppressive factors that confer tolerogenic properties ^28^. One major factor is the enzyme IDO ^28, 70, 71^. This enzyme is involved in the catabolism of the essential amino acid, tryptophan, and the synthesis of kynurenines. Tryptophan is required for T cell proliferation and kynurenines have immunosuppressive properties. Engagement of several receptors, including CD80/CD86, or TLR9 ^72^, participates in active IDO induction. Amino acid withdrawal resulting from IDO enzymatic activity, stimulates the GCN2 kinase in PDC and then prevents IL-6 secretion by PDC ^10^. Moreover, IDO exerts a regulatory function independently of its catabolic activity by participating in TGF-β-signaling pathway ^73^. Receptors expressed by PDC, such as immunoglobulin-like transcript 7 ^74^ or CD303 ^75^ may also inhibit TLR7/9-mediated type I IFN production. In addition, PDC can produce, under certain circumstances, high levels of immunosuppressive cytokines, such as IL-10 ^20, 27^ or TGF-β ^66, 76^. In addition, PDC participate in the maintenance of immune tolerance *via* the induction and/or expansion of regulatory T cells (please refer to Refs# ^7, 76^; this is out of the scope of this review).

When one evokes cellular metabolism, cell survival has to be discussed. IL-3 has been identified as a critical factor for the development and survival of PDC ^77, 78^. This cytokine interacts with the IL-3 receptor associating two chains, the common chain CD131 and the IL-3Rα chain (CD123) that is highly expressed by PDC. Signaling through this receptor involves Janus kinase 2 (JAK2), Src kinases, transcription factors STAT3/STAT5 (signal transducer and activator of transcription 3/5) and Akt ( Figure 1) ^79^. Another cytokine that shares the common chain CD131 and influences PDC survival with nearly the same signaling pathway is GM-CSF ( Figure 1) ^78^. Finally, PDC express IFNAR, the membrane receptor for type I IFN (IFN-α or IFN-β) that consists of two subunits, IFNAR1 and IFNAR2. Engagement of this receptor by its ligand activates JAK1 (associated with IFNAR1) and tyrosine kinase 2 (associated with IFNAR2) that phosphorylate and activate STAT1 and STAT2, respectively ( Figure 1) ^79, 80^.

### 2.4. Implications of PDC in diseases

Before discussing the role of immunometabolism, the role of PDC in beneficial and detrimental immune responses will be briefly detailed. As type I IFN producing cells, PDC play a major beneficial role in antimicrobial immune responses ^7^. However, uncontrolled IFN-α production in acute viral infection may be detrimental to the host. Moreover, high levels of type I IFN released by PDC may be detrimental in chronic inflammatory or autoimmune diseases ^7, 64, 81– 84^. This is the case of systemic lupus erythematosus (SLE) ^62– 64^, type 1 diabetes ^82^, and psoriasis ^51, 84^. Furthermore, PDC may participate in inflammatory autoimmune disorders ( *i.e*., systemic sclerosis ^83^ or autoimmune vasculitis ^85^) *via* the secretion of other pro-inflammatory cytokines than type I IFN ^85^ or chimiokines ^83^. Since PDC infiltrate inflamed tissues, they may release pro-inflammatory factors participating in the amplification of diseases. Therefore, PDC have been reported to infiltrate acute graft- *versus*-host disease lesions, including gastro-intestinal ^86^ and cutaneous ^87^ lesions. PDC emerge as cells present in atherosclerotic plaques and may play a role in atherosclerosis ^79, 88^. Atherosclerotic plaques are enriched in lipids. This may modify the lipid metabolism of infiltrated PDC by the uptake of lipid-enriched lipoproteins or oxidized lipoproteins, and subsequently PDC functions (see section 3.3.3). Finally, insufficient or exhausted production of IFN-α during chronic viral infections (e.g., chronic hepatitis C virus or HIV) has also been reported ^89, 90^. Furthermore, as stated before, PDC have been shown to infiltrate tumors ^33– 40^ and the presence of infiltrating PDC have been associated with a poor prognosis in some tumors ^35, 36, 39, 40^. Defective type I IFN production by tumor-infiltrating PDC has been identified as one potential mechanism explaining tumor progression ^34, 35, 38, 91, 92^. Thus, the metabolism of PDC may be pharmacologically modified in order to restore type I IFN production. The pathological microenvironment in which PDC are present may impact on PDC metabolism and subsequently on their functions. This is particularly true for tumor-infiltrating PDC, since metabolic dysregulation is a common and well-recognized feature of cancer ^93– 95^ . Today, this has been mainly studied in infiltrating T cells and macrophages.

## 3. The influence of the metabolism on innate immune functions of plasmacytoid dendritic cells

### 3.1. PDC innate immune functions and mTOR signaling

The kinase mTOR is a key regulator of different biological processes, including metabolism ^8, 9^. This is a serine/threonine protein kinase that senses and integrates signals (such as nutrients and oxygen) originating from the extracellular milieu, as well as intracellular signals ^96^. In fact, mTOR is the catalytic subunit of two different complexes, mTORC1 and mTORC2. As mentioned briefly in the Introduction, mTORC1 is connected with several metabolic pathways. Indeed, mTORC1 promotes glycolysis through hypoxia-inducible factor 1α (HIF-1α) ^9^, as well as cholesterol and fatty acid synthesis using TCA cycle intermediates through a pathway involving sterol regulatory element-binding proteins (SREBP) and the nuclear receptor, peroxisome proliferator-activated receptor (PPAR) γ ^9^. Cholesterol and fatty acids are used as “building blocks” ^9^ for complete maturation of endoplasmic reticulum (ER) and Golgi apparatus. Both organelles can promote the transport of pro-inflammatory cytokines within the cell that precedes their secretion ^9^. In addition, mTORC1 can have a negative effect on mitochondrial OXPHOS by inducing the expression of type I IFN and production of nitric oxide, which subsequently promotes aerobic glycolysis ^9^. This has been well described in macrophages ^9^.

**Figure 2.**
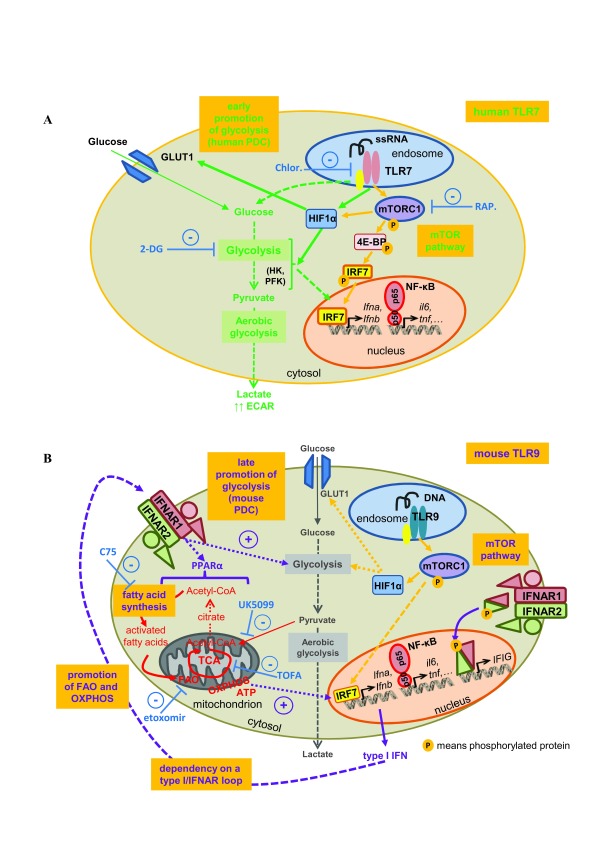
Metabolic changes in plasmacytoid dendritic cells (PDC) providing energy and affecting their innate immune functions: glycolysis *versus* fatty acid oxidation coupled with OXPHOS. ( **A**) The endosomal TLR7 pathway in human blood-derived PDC promotes early glycolysis (within minutes following TLR7 triggering; green font and green arrows), as attested by increased ECAR (extracellular acidification rate; a reflection of lactate excretion). This implicates the HIF-1α molecule that increases the GLUT1 glucose transporter expression allowing extracellular glucose entry. HIF-1α stimulates some enzymes involved in glycolysis (HK or PFK). Glycolysis in human PDC is required for TLR7-induced type I IFN production. A potential link with the activation of the mTORC1 complex (orange arrows) can be seen, since this complex is activated by the endosomal TLR7 pathway and induces HIF-1α in human PDC. Inhibitors of mTORC1 (RAP.), of TLR7 signaling (chlor.), and of glycolysis (2-DG) are written in blue font. All these inhibitors block TLR7-induced type I IFN production. ( **B**) The TLR9 pathway in mouse bone marrow-derived PDC promotes late glycolysis (after 24 hours) (grey font and grey arrows) *via* a type I IFN/IFNAR loop (violet arrows). Through this loop, the TLR9 pathway also promotes fatty acid synthesis, FAO coupled with OXPHOS to generate ATP in a PPARα-dependent mechanism (violet font and violet arrows). This TLR9 pathway implicates the activation of mTORC1 in mouse PDC (orange arrows, as depicted in Figure 2A). Specific inhibitors of fatty acid synthesis (C75), pyruvate entry in the mitochondrion (UK5099), TCA cycle (TOFA) or FAO (etoxomir) are written in blue font and have been used to demonstrate the promotion of fatty acid synthesis, FAO and OXPHOS in TLR9-induced type I IFN production, respectively. For more details, please refer to the main text. Abbreviations (not defined in the main text): HK, hexokinase; PFK, phosphofructokinase; RAP., rapamycin; chlor., chloroquine.

Concerning PDC innate immune functions, mTOR plays an important role in type I IFN production ^97^. The TLR9 ligand, CpGA, stimulates the rapid phosphorylation of mTOR and its downstream targets, the p70 ribosomal S6 kinase 1 and the eukaryotic translation initiation factor 4E-binding protein (4E-BP) ^97^. Thus, mTOR is involved in the TLR9-induced type I IFN signaling pathway ( Figure 2). Using the mTORC1 inhibitor rapamycin, the same authors demonstrated that TLR7/9-mediated production of type I IFN is inhibited in both human and mouse PDC ^97^. These data have been confirmed *in vivo* using live attenuated viral yellow fever vaccine ^97^. The virus responsible for yellow fever is an ssRNA virus and TLR7 recognizes ssRNA ^48^. Rapamycin may act at two levels: *i*) by inactivating *via* the inhibition of 4E-BP phosphorylation the nuclear translocation of IRF7 required for type I IFN gene transcription; and *ii*) by blocking the formation of the TLR9-MyD88 complex *via* the p70 ribosomal S6 kinase 1 ^97^. In addition to type I IFN production, the inhibition of mTORC1 reduces TLR7/9-induced TNF and IL-6 production by human and mouse PDC ^97^. Overall, mTOR signaling is involved in the TLR9-induced type I IFN signaling pathway and blocking mTORC1 inhibits TLR7/9-stimulated secretion of pro-inflammatory cytokines ( *i.e.,* type I IFN, TNF and IL-6) by PDC. Thus, the use of mTOR inhibitors may block the detrimental pro-inflammatory functions of PDC in inflammatory disorders, but may also prevent the beneficial antimicrobial response of PDC.

Lastly, mTORC1 stimulates glycolysis in innate immune cells, at least *via* HIF-1α induction, but also through the increased expression of the glucose transporter 1 (GLUT1) at the cell surface ( Figure 2) ^9^. GLUT1 enhances glucose uptake from the extracellular milieu ^9^. Thus, the implication of mTOR signaling pathway in the response of PDC to TLR9 ligand ^97^ suggests that glycolysis may be triggered in the cells. This will be discussed in the next section.

### 3.2. PDC innate immune functions and glycolysis

The glycolytic pathway (also known as glycolysis) involves the uptake of extracellular glucose ( *i.e*., present in the microenvironment) and its conversion in the cytosol to generate pyruvate ^2^. Most pyruvate is then excreted from the cells as lactate after a process called aerobic glycolysis. This is a relatively inefficient pathway for the generation of energy ( *i.e*., cellular ATP) compared to the TCA cycle coupled to OXPHOS ^98^. The preferential use of glycolysis *versus* the TCA cycle and OXPHOS depends on oxygen availability ^2, 98^. Glycolysis is favored during hypoxia, a situation encountered, for instance, in joints during rheumatoid arthritis or in the inflamed colon during Crohn’s disease ^98^ - PDC can be present in both inflamed tissues ( *i.e*., the synovial fluid ^99– 101^ and the colon ^86, 102^). Regions of hypoxia are also present in solid tumors ^95^ infiltrated by PDC. This energy provided by glycolysis may represent the resources necessary for cytokine secretion, since PDC does not proliferate in the same way as adaptive cells ( *i.e*., T cells). However, PDC become highly secretory upon activation ^7^. Finally, growth factors triggering PI3K and MAPK in their signaling pathway promote, in theory, the cellular use of glycolysis ^2^. This may be the case when PDC are stimulated by TLR7/9 ligands (see previous section).

The exposure of human PDC to two different ssRNA viruses, influenza virus (Flu) and Rhinovirus (RV) – triggering the TLR7 pathway *via* ssRNA recognition ^48^ – activates HIF-1α ^4^, a major regulator of metabolism ( Figure 2A). Indeed, HIF-1α is critical for glycolysis to generate ATP, since it induces the expression of different glycolytic enzymes, such as hexokinase and phosphofructokinase ^98^. In addition to HIF-1α activation, the TLR7 agonist gardiquimod, as well as Flu and RV, induces early glycolysis (within minutes) in human PDC, as attested by elevated extracellular acidification rate (ECAR; a reflection of lactate secretion in extracellular milieu, an indicator of glycolysis in real time) and elevated rates of lactate production ^4^. Moreover, the inhibition of glycolysis by 2-deoxyglucose (2-DG; a glycolytic inhibitor) impairs ssRNA virus- or TLR7 ligand-induced type I IFN by PDC, as well as the upregulation of HLA-DR, CD80 and CD86 at PDC cell surface ^4^. Furthermore, 2-DG inhibits the increase of *IFNA*, *CD80* and *CD86* mRNA induced by exposure to Flu ^4^. This suggests that glycolysis induced by the TLR7 pathway regulates these genes at the transcriptional level. The involvement of the TLR7 pathway in glycolysis was supported by the use of chloroquine, known to disrupt endosomal acidification required for TLR7 signaling ^103^. Chloroquine treatment inhibits the lactate production by PDC in response to Flu or gardiquimod ^4^. Overall, ssRNA viruses enhance glycolysis in human PDC *via* the TLR7 pathway ( Figure 2A). This finding was confirmed *in vivo*, since viral infection using live attenuated influenza vaccine increases glycolysis in *ex vivo* isolated human PDC and correlates with IFN-α production by these cells ^4^.

These data contrast with a recent report showing that mouse PDC activation in response to the TLR9 ligand, CpGA, is not accompanied by a rapid change in ECAR ( *i.e*., during the first 150 minutes). This contrasts with data obtained in the same experiments using cDC – as a control of PDC – stimulated by either lipopolysaccharide, Poly(I:C) or CpGA ^5^. However, glycolysis is detected late (24 hours) in TLR9-stimulated mouse PDC ^5^. Moreover, the authors also studied the role of the TLR7 pathway in mouse PDC, but not extensively. Again, they found a delayed activation of glycolysis after stimulation of mouse PDC with the TLR7 agonist imiquimod ^5^. Thus, whether the origin of PDC (human ^4^ *versus* mouse ^5^) or their source (sorted from peripheral blood mononuclear cells ^4^ *versus* sorted from FLT3 ligand-stimulated bone marrow cultures ^5^) may explain this discrepancy remains to be determined. Another difference between these two studies lies in the direct effect of IFN-α on PDC metabolism. Treatment of human PDC with IFN-α is not sufficient to induce PDC lactate efflux ( *i.e*., glycolysis) and IFN-α/β receptor (IFNAR) blockade does not affect PDC lactate efflux induced by Flu infection ^4^. This suggests that type I IFN does not regulate in an autocrine/paracrine manner Flu-induced early glycolysis in human PDC. In contrast, this autocrine/paracrine loop involving type I IFN and its receptor may play a significant role in TLR-induced glycolysis in mouse PDC ( Figure 2B) ^5^. Nevertheless, all these data support that glycolysis plays a role as a source of energy in the production of type I IFN, pro-inflammatory cytokines (IL-6 and TNF) and costimulatory molecule upregulation by PDC in response to TLR7/9 activation. The modulation of this metabolic pathway may limit uncontrolled pro-inflammatory cytokines by PDC in pathological situations or may restore type I IFN production in chronic infectious diseases or in solid tumors.

### 3.3. PDC innate immune functions and lipid metabolism

In this section, we will discuss fatty acid metabolism, including fatty acid oxidation (FAO; also known as mitochondrial β-oxidation) and fatty acid synthesis, as well as cholesterol metabolism. Lipid metabolism is regulated by many key enzymes. Some of these enzymes involved in *de novo* lipid synthesis are controlled by lipid-activated nuclear receptors, such as liver X receptor (LXR) or PPAR. The genes coding for these enzymes are thus called LXR or PPAR target genes, respectively. Among these LXR or PPAR target genes, one may cite *FASN* coding for fatty acid synthase ^104– 106^. These LXR or PPAR target genes code not only for enzymes involved in lipid metabolism, but also for transcription factors and transporters, and regulate also glucose or amino acid metabolism. This is the case of the transcription factors, SREBP, or of glucose or cholesterol transporters involved in nutrient entry (e.g., the GLUT1 glucose transporter) or efflux, such as ATP binding cassette (ABC) transporters A1 and G1 (ABCA1 and ABCG1, respectively) involved in cholesterol efflux ^2, 104, 105, 107, 108^. PPAR and LXR are both mainly found associated with retinoid X receptor (RXR) to form a heterodimer. Considered as permissive heterodimer receptors, they can be activated by the ligands of each partner (e.g., PPAR or RXR ligands; LXR or RXR ligands) ^106, 108^. While the involvement of these nuclear receptors in innate immune responses is well described for macrophages and cDC ^108^, few data are available for PDC. Here, we will discuss how lipid metabolism modulates PDC innate immune functions and how PDC activation by TLR ligands or other stimuli modifies lipid metabolism.

***3.3.1. Fatty acid oxidation***. The FAO pathway allows the conversion of fatty acids into numerous products in the mitochondria. These products, such as acetyl-CoA, NADH (nicotinamide adenine dinucleotide dehydrogenase) and FADH _2_ (the fully reduced form of flavin adenine dinucleotide [FAD]), can be used in the TCA cycle and the electron transport chain to generate energy ^2^. As discussed before, normoxia supports the TCA cycle and OXPHOS, while hypoxia *via* HIF-1α activation followed by the induction of glycolytic enzymes leads to glycolysis ^2, 98^. The TCA cycle coupled to OXPHOS is the major metabolic pathway used by most quiescent or non-proliferating cells ^2^.

A recent well-received manuscript reports that FAO and mitochondrial OXPHOS play a critical role in murine PDC activation by the TLR9 pathway ( Figure 2B) ^5^. This is particularly well demonstrated for type I IFN production by these cells ^5^. Mouse PDC stimulated by the TLR9 ligand, CpGA, exhibit an increase of basal oxygen consumption rate (OCR) and spare respiratory capacity (SRC) ^5^. Both increased basal OCR and SRC are indicators of FAO. To directly demonstrate that CpGA increases FAO, the authors used etomoxir, an irreversible inhibitor of carnitine palmitoyl transferase I ^2^. This enzyme is responsible for the entry of activated fatty acids ( *i.e*., medium-chain and long-chain fatty acids conjugated with carnitine) into mitochondria for FAO ^2^. Etomoxir inhibits both the increase of basal OCR and SRC induced by TLR9 ligand stimulation. Moreover, etomoxir limits the production of IFN-α and pro-inflammatory cytokines (TNF-α and IL-6) by PDC in response to CpGA stimulation. This inhibition of TLR9 activation by etomoxir also prevents the upregulation of CD86 expression at PDC cell surface ^5^. Overall, TLR9-induced mouse PDC activation is accompanied by an increased FAO, and stimulation of this metabolic pathway is required for pro-inflammatory cytokine secretion and PDC maturation ( *i.e*., CD86 upregulation).

An increase of basal OCR and SRC attesting for FAO has also been observed after the activation of mouse PDC by the TLR7 agonist imiquimod. Treatment of mouse PDC by etomoxir inhibits imiquimod-induced IFN-α production ^5^, suggesting that FAO is also required for type I IFN production in response to TLR7 ligand. Implication of FAO *in vivo* was assessed using etomoxir and mice infected with the ssRNA lymphocytic choriomeningitis virus (LCMV). Etomoxir-treated and LCMV-infected mice exhibit reduced circulating IFN-α 3 days after infection and significantly more LCMV are detected in the liver and spleen of etomoxir-treated *versus* untreated infected mice ^5^. This demonstrates the *in vivo* relevance of these data.

Changes in basal OCR are not detected at early time points after murine PDC activation by CpGA, but this requires new gene transcription. This may suggest that IFN-α production and IFNAR signaling induced by CpGA stimulation may be responsible for these changes in PDC metabolism. Indeed, treatment with IFN-α alone is sufficient to induce increased FAO in mouse PDC ^5^. Thus, increased FAO induced by CpGA stimulation in mouse PDC are therefore the results of an autocrine or paracrine loop involving the type I IFN signaling pathway.

One goal of the FAO pathway is to generate energy, by the production of a high number of ATP molecules ^2^. This occurs in fact by fueling OXPHOS ^2^. To definitively demonstrate the implication of energy provided by FAO coupled to OXPHOS in type I IFN-induced mouse PDC activation, ATP was quantified in response to IFN-α and different inhibitors were used ^5^. Metabolic reprogramming of mouse PDC induced by IFN-α leads to enhanced ATP availability ^5^. The quantity of ATP in response to CpGA activation is significantly reduced by the inhibition of either FAO (using etomoxir), pyruvate import into mitochondria required for the TCA cycle (using UK5099) or fatty acid synthesis (using tall oil fatty acid [TOFA]) ^5^. This confirms that type I IFN stimulation of mouse PDC generates significant amounts of ATP *via* the FAO pathway. This pathway fuels OXPHOS in this setting and is itself fueled by fatty acid synthesis, as demonstrated by the use of the inhibitor TOFA ( Figure 2B).

The pathways responsible for the changes in metabolism induced by type I IFN in mouse PDC were studied by an unbiased RNA-seq based approach ^5^. This analysis shows that OXPHOS is the major network induced by type I IFN, and FAO is connected to this network. Furthermore and surprisingly, this analysis reveals also a PPARα gene signature ^5^. While PPARγ is expressed in macrophages and cDC ^108^, the PPARα isoform is mainly and highly expressed in metabolic active tissues, such as the liver or brown adipose tissues ^106^. After having confirmed that the PPARα isoform is expressed by bone marrow-derived and splenic mouse PDC, a PPARα antagonist, GW6471 was used ^5^. GW6471 blocks both IFN-α production and the increase of basal OCR in response to CpGA activation. Furthermore, increased basal OCR and SRC in mouse PDC are observed after incubation with a PPARα agonist gemfibrozil, as well as with the combined PPARα and PPARγ agonist, muraglitazar ^5^. Overall, this indicates that PPARα is involved in FAO and OXPHOS induced by CpGA activation ( Figure 2B). This PPARα pathway in PDC functions will be discussed later in this review together with the other lipid-activated nuclear receptor, LXR (see the following two sections).

***3.3.2. Fatty acid synthesis***. The fatty acid synthesis pathway allows cells to generate lipids that are necessary for cellular growth and proliferation ^2^. Fatty acid synthesis is performed in the cytosol, and it uses citrate from the TCA cycle and exported from the mitochondria into the cytosol to generate fatty acids ( Figure 2B). As mentioned above, *de novo* fatty acid synthesis is dependent on key enzymes, such as FASN, that are controlled by either the mTOR signaling pathway ^9^, LXR ^104, 105^ or SREBP-1c ^2, 109^. Fatty acids generated by this synthesis can be used to fuel mitochondrial FAO ^2^, to activate PPAR nuclear receptors ^107^, or can be condensed with glycolysis-derived glycerol to produce triacylglycerol and phospholipids ^2^. These latter two are key components of many cellular structures, such as the cell membrane, ER or Golgi apparatus ^2, 9^.

The inhibition of the fatty acid synthesis using two different inhibitors (TOFA, an inhibitor of acetyl-CoA carboxylase; C75, an inhibitor of FASN) ^2^ prevents the increase of IFN-α, TNF-α, and IL-6 production by mouse PDC in response to CpGA stimulation ^5^. As previously demonstrated for FAO and OXPHOS, fatty acid synthesis induced by the TLR9 pathway implicates a type I IFN/IFNAR loop ^5^. Moreover, using the TOFA inhibitor before measuring both the quantity of ATP in response to CpGA activation and the expression of PPARα target genes, *Acadl* and *Pltp* in CpGA-activated mouse PDC ^5^ allowed the authors to conclude that fatty acid synthesis induced in mouse PDC rather fuels FAO and OXPHOS than provides lipid ligands for PPARα activation. It is well described that the natural ligands of PPARα include different fatty acids, as well as numerous fatty acid derivatives and compounds with structural resemblance to fatty acids, such as acyl-CoAs, or oxidized fatty acids ^107^. Here in PDC, fatty acids synthetized in response to the TLR9 signaling pathway do not seem to directly stimulate PPARα by providing PPARα ligands. One may hypothesize that, as reported for lipid-activated nuclear receptors ^104, 106^, activation of PPARα by TLR9 ligand may interfere with transcription factors ^3, 106^ that are critical for pro-inflammatory cytokine secretion by PDC (see section below).

**Figure 3.**
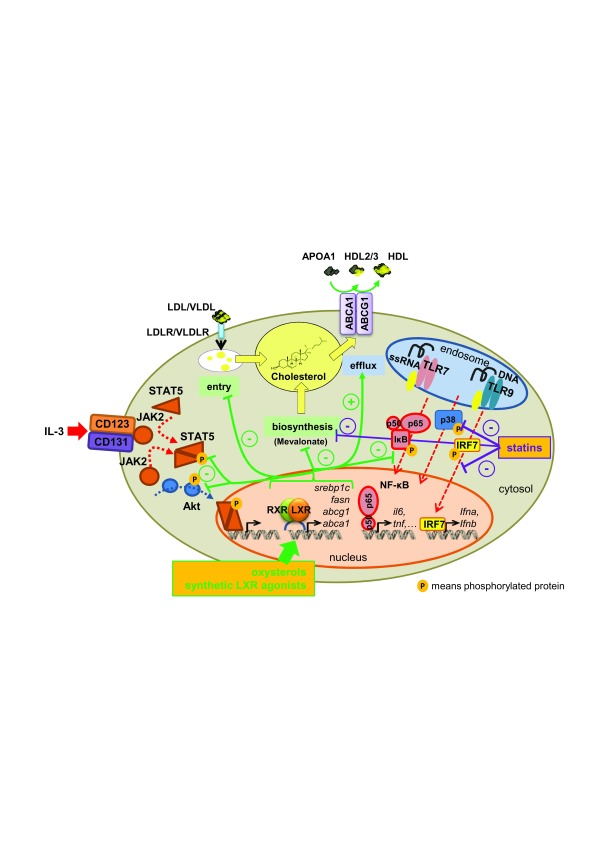
Cholesterol metabolism controls plasmacytoid dendritic cell (PDC) innate functions. Activation of the LXR pathway by “physiological” oxidized cholesterol derivatives (oxysterols) or synthetic LXR agonists induces the decrease of PDC intracellular cholesterol content by stimulating cholesterol efflux through ABCA1 cholesterol transporter, inhibiting cholesterol entry by decreasing LDL or VLDL receptor (LDLR and VLDLR, respectively) expression and inhibiting *de novo* cholesterol biosynthesis (also known as the mevalonate pathway). Cholesterol efflux *via* ABCA1 requires cholesterol acceptors, such as APOA1, and immature HDL (HDL2/3) to generate mature HDL that transport cholesterol towards the liver. Activation of this LXR pathway inhibits TLR7-induced NF-κB activation and phosphorylation of STAT5 and Akt in response to IL-3 stimulation (green font and green arrows). Inhibition of cholesterol biosynthesis by statins (violet font and arrows) inhibits TLR7/9-induced IRF7 translocation in the nucleus, as well as phosphorylation of p38 kinase, and consequently the production of type I IFN. For more details and abbreviations, please refer to the main text.

***3.3.3. Cholesterol metabolism***. Cholesterol is one of the major constituents of lipid rafts together with glycosphingolipids ^110^. Cellular cholesterol content results from cholesterol uptake and biosynthesis through the mevalonate pathway, while its elimination from cells is mediated by cholesterol efflux. Cholesterol uptake involves plasma lipoproteins (mainly low density lipoprotein [LDL] and very low density lipoprotein [VLDL]) after interactions with their specific receptors, LDLR and VLDLR, respectively. Cholesterol efflux is mainly mediated by specific transporters, ABCA1 and ABCG1, in association with extracellular cholesterol acceptors, including apolipoproteins (APO) APOA1 and APOE, or lipoprotein particles (e.g., nascent high density lipoprotein [HDL] or HDL2) ( Figure 3) ^111^. ABCG1 is rather localized within the cell and seems to move sterols between intracellular compartments ^106^. ABCA1 is more dedicated to extrude cholesterol derivatives outside the cell ^106^. Cholesterol regulates critical cellular functions, including plasma membrane formation and fluidity, allowing the clustering of receptors into lipid rafts for efficient signaling ^110^. These latter functions are implicated in signaling pathway regulation. Localization of signaling complexes within the lipid rafts is critical for certain receptors.

Cholesterol homeostasis is regulated at least by LXR. These nuclear receptors are expressed as two isoforms, including the ubiquitous LXRβ isoform and LXRα, which exhibits an expression restricted to cells with high cholesterol turnover (e.g., macrophages) ^104, 108, 112^. The LXR pathway is activated by intermediates from the cholesterol biosynthesis (e.g., desmosterol), endogenous oxidized cholesterol derivatives (called oxysterols, such as 22(R)-hydroxycholesterol [22RHC]), and synthetic agonists (e.g., T0901317 or GW3965) ^104^. LXR activation upregulates the expression of several genes involved in cholesterol homeostasis ( *i.e.*, LXR target genes), including: *ABCA1*, *ABCG1* ^105^, and *APOE* (related to cholesterol efflux) ^106, 113^, as well as the ‘inducible degrader of the low-density lipoprotein receptor’ (IDOL), preventing cholesterol uptake through LDLR/VLDLR degradation ^114^. Overall, these mechanisms triggered by LXR activation participate in the decrease of intracellular cholesterol content. Again, the LXR pathway has been studied extensively in the regulation of macrophage functions, including cholesterol homeostasis and inflammatory responses, as well as apoptotic cell uptake (a process called efferocytosis) ^108^. However, the role of cholesterol and LXR begins only to be deciphered in PDC innate functions.

Inhibition of the mevalonate pathway ( *i.e*., *de novo* cholesterol biosynthesis) by statins (simvastatin and pitavastatin) blocks TLR7- and TLR9-induced type I IFN production by human PDC ( Figure 3) ^115^. This has been demonstrated with blood-derived PDC obtained from healthy donors, as well as from patients with SLE ^115^. Statin treatment also inhibits TNF secretion by human PDC in response to either TLR7 (loxoribine) or TLR9 (CpGA) ligands ^115^. These data have been confirmed *in vivo* using ssRNA Sendai virus ^115^. Inhibition of *de novo* cholesterol synthesis by statins in PDC interferes with the p38 MAPK pathway, Akt and nuclear translocation of IRF7 ( Figure 3) ^115^. Finally, the inhibitory effect of statins has been tested *in vivo* and on mouse PDC, since treatment of C57BL/6 mouse with statins before triggering type I IFN production by ssRNA poly(U) injection decreases circulating IFN-α ^115^. Thus, the inhibition of cholesterol synthesis in PDC is associated with an anti-inflammatory response. This confirms the global anti-inflammatory effects of statins ^116^.

Recently, we reported that the LXRβ isoform is expressed in human PDC ^6^, as well as in mouse PDC (unpublished study, Ceroi A, Bonnefoy F, Angelot-Delettre F and Saas P), and that this LXR pathway is fully functional ^6^. Using freshly blood-isolated human PDC and a PDC cell line CAL-1, a functional LXR pathway has been demonstrated, as attested by increased LXR target gene expression in response to three different LXR agonists (two synthetic agonists and an oxysterol, 22RHC that represents a more physiological LXR ligand). The activation of the LXR pathway in PDC reduces the pro-inflammatory cytokine secretion (IL-6 and TNF-α) induced by TLR7 triggering ^6^. Moreover, these data obtained in human PDC from healthy donors ^6^ and in leukemic PDC ^117^ demonstrate that the LXR pathway interferes with TLR7-induced NF-κB activation at different levels, including a transcriptional repression of p65 NF-κB subunit and a reduced phosphorylation of this NF-κB subunit ( Figure 3) ^6, 117^. Pretreatment of leukemic PDC with synthetic LXR agonists also reduces Akt and STAT5 phosphorylation in response to IL-3 ( Figure 3) ^117^. LXR stimulation in the PDC cell line CAL-1 increases cholesterol efflux *via* the upregulation of cholesterol transporters, such as ABCA1 ( Figure 3) ^117^. Although the cholesterol efflux was only tested using this CAL-1 PDC cell line ^117^, upregulation of cholesterol transporters at mRNA and protein levels was also observed in human blood-derived PDC treated with LXR agonists ^6^. Stimulation of cholesterol efflux by the addition of a cholesterol acceptor, APOA1, amplifies the effects of LXR activation in leukemic PDC, including inhibition of the IL-3 signaling pathway (Akt and STAT5 phosphorylation) and cell survival ( Figure 3) ^117^. This confirms the previous data using statins ^115^, and suggests that modifying cholesterol homeostasis in PDC can be useful to limit their detrimental role in pathological situations. Alteration of PDC survival after modification of intracellular cholesterol content using either statins ^115^ or LXR agonists ^117^ is only detected at highest concentrations (100 µM). In addition, LXR activation in human PDC also increases microparticle internalization *via* the phosphatidylserine receptor (PtdSerR), BAI-1 ^6^. This contrasts with data obtained in macrophages in which LXR stimulation induces another PtdSerR, called Mer-TK (Mer tyrosine kinase) ^118^. Nevertheless, this suggests that stimulation of PDC *via* the LXR pathway may improve their capacity to eliminate circulating pro-inflammatory microparticles. Triggering LXR pathway using LXR agonists before exposure to pro-inflammatory endothelial-derived microparticles prevents NF-κB activation and pro-inflammatory cytokine production by human PDC ^6^. This sustains an anti-inflammatory role of LXR agonists.

A discrepancy exists concerning type I IFN production after inhibition of cholesterol biosynthesis (using statins ^115^) and the massive decrease of intracellular cholesterol content (using LXR agonists ^6, 117^). Inhibition of type I IFN has been reported after statin treatment ^115^, but not after cholesterol deprivation ^6^. This may be due to a compensatory mechanism, since massively decreasing the pool size of synthesized cholesterol alone induces spontaneous type I IFN production associated with an antiviral immunity in bone marrow-derived macrophages ^119^. This response occurs *via* the cGAS/STING/TBK1/IRF3 pathway ^119^. Thus, LXR agonists could inhibit TLR7/9-mediated type I IFN by interfering with the mevalonate pathway ( *i.e*., *de novo* cholesterol synthesis), as statins did. Simultaneously, LXR agonists could stimulate type I IFN production as a result of a massive decrease in the intracellular cholesterol pool size. This compensatory mechanism could explain why IFN-α production is unaffected after LXR agonist treatment of PDC. Another difference between the effect of statins and LXR agonists lies in the inhibition of IRF7 (but not of NF-κB phosphorylation by statins) ^115^, whereas the activation of the LXR pathway in PDC blocks NF-κB activation *via* several mechanisms ^6, 117^ (see above). Overall, the manipulation of cholesterol metabolism in PDC can be proposed to limit their pro-inflammatory functions.

## 4. Conclusions

Here, we summarize data currently available showing that several metabolic pathways are triggered in PDC by different stimuli, including pro-inflammatory signals (e.g., TLR7/9 ligands or endothelial-derived microparticles), as well as anti-inflammatory signals (e.g., platelet-derived microparticles). These pathways comprise: the mTOR signaling pathway, glycolysis, FAO coupled to OXPHOS, fatty acid synthesis and cholesterol metabolism ( Table 1). All of these pathways are connected together, and they are globally necessary for efficient type I IFN production by PDC in response to TLR7/9-mediated activation. Few data are available, but it seems that induction of pro-inflammatory cytokines (e.g., TNF, IL-6 and IL-8) and costimulatory molecules ( *i.e*., CD80 or CD86) also need most of these metabolic pathways ( Table 1). On the contrary, alteration of cholesterol metabolism associated with decreased intracellular cholesterol content either after inhibition of *de novo* cholesterol synthesis or LXR activation inhibits the pro-inflammatory functions of PDC ( Table 1). These data suggest that pharmacological manipulation of the host metabolism may be useful to reprogram altered PDC immune functions.

**Table 1.** Innate immune functions of plasmacytoid dendritic cells are modulated by or dependent on metabolic pathways.

Metabolic pathways	PDC	Stimulus used to stimulate PDC	Pharmacological agent used	Affected innate immune functions	References
**mTOR signaling**	human blood-sorted or mouse spleen-sorted PDC	CpGA (TLR9), attenuated yellow fever vaccine	rapamycin (inhibitor of mTORC1)	Type I IFN, IL-6, TNF-α	97
**Glycolysis**	human blood-sorted PDC	Gardiquimod (TLR7), influenza virus and Rhinovirus	2-deoxyglucose (inhibitor of glycolysis)	Type I IFN, expression of HLA-DR, CD80, and CD86	4
**Fatty acid oxidation** **(FAO) coupled to** **OXPHOS**	mouse PDC (sorted from FLT3 ligand-stimulated bone marrow cultures)	CpGA (TLR9)	etoxomir (inhibitor of carnitine palmitoyl transferase I) *	Type I IFN, TNF-α, IL-6, expression of CD86	5
	mouse PDC (sorted from FLT3 ligand-stimulated bone marrow cultures)	imiquimod (TLR7), LCMV	etoxomir	Type I IFN	5
	mouse PDC (sorted from FLT3 ligand-stimulated bone marrow cultures)	CpGA (TLR9)	GW6471 (inhibitor of PPARα)	Type I IFN	5
**Fatty acid** **synthesis**	mouse PDC (sorted from FLT3 ligand-stimulated bone marrow cultures)	CpGA (TLR9)	TOFA (inhibitor of acetyl-CoA carboxylase), C75 (inhibitor of FASN)	Type I IFN, IL-6, TNF-α	5
**Cholesterol** **metabolism**	human blood-sorted PDC	CpGA (TLR9), loxoribine (TLR7), Sendai virus	simvastatin or pitavastatin (inhibitors of *de novo* cholesterol synthesis)	Type I IFN, TNF-α	115
	mouse PDC	ssRNA Poly(U)	simvastatin or pitavastatin	IFN-α	115
	human blood-sorted PDC	R848 (TLR7), endothelial-derived microparticles	GW3965, T0901317, 22RHC (LXR agonists)	TNF-α, IL-6	6

*This agent inhibits the entry of activated fatty acids into mitochondria for FAO. For abbreviations, please refer to the main text.

Since cellular metabolism is highly dependent on the microenvironment (oxygen availability and nutrients), changes in the local tissue microenvironment may modulate PDC innate immune functions. This modulation of metabolism may result from exogenous metabolites that diffuse passively or through transporters into the PDC. Among these metabolites, one may find ligands of lipid-activated nuclear receptors, such as LXR or PPAR ^3, 108^.

Microenvironment and metabolic pathways may be modulated or controlled by microbiota. A recent study analyzed germ-free mice mono-colonized with each of the 53 human-resident bacterial species and the consequences of each bacterium on different immune cell subsets ^120^. Among these 53 bacteria, some bacteria were identified as modifying PDC frequencies in the colon and the small intestine ^120^. Among the genes for which their expression was correlated with PDC frequencies, the authors identified IFN-inducible signature transcripts, but also transcripts involved in lipid and protein metabolic pathways. Moreover, the *Hif1a* transcript coding for the metabolism regulator HIF-1α was also associated with PDC frequencies ^120^. This suggests a connection between PDC frequencies in the gastro-intestinal tract and metabolic pathways and nutrients/metabolites provided by microbiota.

We discussed above the connection of metabolism with epigenetic regulation. While few data are available concerning epigenetic regulation of PDC innate immune functions, it has been shown that the inhibitor of histone deacetylase, valproic acid, alters human PDC functions, including the production of pro-inflammatory cytokines (IFN-α TNF and IL-6) in response to TLR9 ligand, CpGA ^121^. Thus, it remains to be determined how metabolism may regulate epigenetic modification of DNA and histones in PDC.

Lastly, acute perturbations in intracellular lipid content, *via* for instance LXR activation, may also influence cell proliferation and survival by inducing significant ER stress ^106^. This cellular organelle is responsible for protein folding. The result of this ER stress is an accumulation of unfolded proteins. This pathway leads also to the expression of the transcription factor X-box binding protein 1 (XBP1), which induces lipid synthesis. XBP1 has been shown to regulate cDC infiltrating ovarian tumors; accumulation of lipids in the tumor-infiltrating cDC following ER stress and XBP1 activation reduces their ability to present antigens, and thus impairs anti-tumor T-cell responses ^122^. Whether this may occur in infiltrating PDC remains to be analyzed. This may concern tumor-infiltrating PDC or PDC present in inflamed tissues. Nevertheless, PDC have been shown to express XBP1 ^123, 124^. This factor can be targeted with bortezomib, which disturbs ER homeostasis ^124^. This can be an additional way related to metabolism to block pro-inflammatory PDC functions. In conclusion, a better understanding of PDC immunometabolism may help to limit the detrimental effect of these cells and increase their beneficial role in the future.
