# Supplementary material for: Recent insights into the implications of metabolism in plasmacytoid dendritic cell innate functions: Potential ways to control these functions
Source: F1000Res. 2017 Jun 22;6:456. Originally published 2017 Apr 10. [Version 2] doi: 10.12688/f1000research.11332.2 (PMC5437952; doi:10.12688/f1000research.11332.2)
Supplement: Peer review history

# Peer review: Referee response for version 2

**DOI:** 10.5256/f1000research.12930.r23752

**Published:** 2017-07-24

**Authors:** Edward J. Pearce, Katarzyna Grzes

**Affiliations:** 1 Department of Immunometabolism, Max Planck Institute of Immunobiology and Epigenetics, Freiburg, Germany; 2 Max Planck Institute of Immunobiology and Epigenetics, Freiburg, Germany

We have read this submission. We believe that we have an appropriate level of expertise to confirm that it is of an acceptable scientific standard.

# Peer review: Referee response for version 2

**DOI:** 10.5256/f1000research.12930.r23750

**Published:** 2017-07-05

**Authors:** Nathalie Bendriss-Vermare

**Affiliations:** 1 Team Caux (French National Institute of Health and Medical Research) INSERM U1052/CNRS 5286 (French National Center for Scientific Research), Cancer Research Centre of Lyon (CRCL), Lyon, France

The authors have rigorously addressed each aspect of my comments. This revised manuscript is now suitable for indexing.

I have read this submission. I believe that I have an appropriate level of expertise to confirm that it is of an acceptable scientific standard.

# Peer review: Referee response for version 1

**DOI:** 10.5256/f1000research.12232.r22647

**Published:** 2017-05-12

**Authors:** Edward J. Pearce, Katarzyna Grzes

**Affiliations:** 1 Department of Immunometabolism, Max Planck Institute of Immunobiology and Epigenetics, Freiburg, Germany; 2 Max Planck Institute of Immunobiology and Epigenetics, Freiburg, Germany

In the review by Saas et al., the authors discussed the collective knowledge of the metabolic status of pDC. Despite the fact that data on this subject are rather scarce, the authors prepared an interesting manuscript. They also included a section about the role and function of pDC which created a more comprehensive source of information.

 Comments:

- Abstract needs more clarity, i.e. “Recent data support the idea that the glycolytic pathway (or glycolysis), as well as lipid metabolism (including both cholesterol and fatty acid metabolism) may impact some innate immune functions of PDC or may be involved in these functions after Toll-like receptor (TLR) 7/9 triggering. Some differences may be related to the origin of PDC (human *versus* mouse PDC or blood-sorted *versus* FLT3 ligand stimulated-bone marrow-sorted PDC).” If the authors want to keep this in the abstract they should probably mention what the differences are.
- Please consider rearranging and shortening the Introduction to avoid repetition.
- When talking about the role of mTOR in pDCs, I think there is no need for a detailed introduction to mTOR – there are many recent authoratitive reviews on this subject. The authors should refer to one of these.  For example, a review by David Sabatini.
- The language of the review can be confusing at time. For example, the following sentence should probably be deleted for the sake of clarity: “After differentiation in the bone marrow, PDC are released into the bloodstream for homing to different lymphoid tissues. Thus, PDC isolated from blood of healthy donors or patients consist in PDC migrating to these tissues.”
- The authors should make an attempt to reference original work rather than other reviews. For example, “Localizations of PDC in other lymphoid organs, such as Peyer’s patches of the gut”. Instead of Li et al. (2011) ^1^, please cite the paper by Contractor et al.(2007) ^2^.

We have read this submission. We believe that we have an appropriate level of expertise to confirm that it is of an acceptable scientific standard, however we have significant reservations, as outlined above.

## References

[1] LiHSGelbardAMartinezGJEsashiEZhangHNguyen-JacksonHLiuYJOverwijkWWWatowichSS: Cell-intrinsic role for IFN-α-STAT1 signals in regulating murine Peyer patch plasmacytoid dendritic cells and conditioning an inflammatory response. *Blood*.2011;118(14) : 10.1182/blood-2011-04-349761 3879-89 10.1182/blood-2011-04-349761 21828128PMC3193265

[2] ContractorNLoutenJKimLBironCKelsallB: Cutting Edge: Peyer's Patch Plasmacytoid Dendritic Cells (pDCs) Produce Low Levels of Type I Interferons: Possible Role for IL-10, TGF , and Prostaglandin E2 in Conditioning a Unique Mucosal pDC Phenotype. *The Journal of Immunology*.2007;179(5) : 10.4049/jimmunol.179.5.2690 2690-2694 10.4049/jimmunol.179.5.2690 17709480

# Response

**Published:** 2017-06-15

**Authors:** Philippe Saas

**Affiliations:** Department of Immunology and Cell Therapy, University of Bourgogne Franche-Comté, INSERM - EFS, France

We thank the reviewers for their constructive comments. We appreciate greatly these comments and we have tried to answer to them. Please find below a point by point response to the reviewers’ comments.

*1. Abstract needs more clarity, i.e. “Recent data support the idea that the glycolytic pathway (or glycolysis), as well as lipid metabolism (including both cholesterol and fatty acid metabolism) may impact some innate immune functions of PDC or may be involved in these functions after Toll-like receptor (TLR) 7/9 triggering. Some differences may be related to the origin of PDC (human versus mouse PDC or blood-sorted versus FLT3 ligand stimulated-bone marrow-sorted PDC).” If the authors want to keep this in the abstract they should probably mention what the differences are.*

We agree with this comment. It is similar to the first comment of reviewer #2. We have deleted the following sentence: “..Some differences may be related to the origin of PDC (human *versus* mouse PDC or blood-sorted versus FLT3 ligand stimulated-bone marrow-sorted PDC).“

*2. Please consider rearranging and shortening the Introduction to avoid repetition.*

We prefer to keep the repetition for readers not involved in the fields of either metabolism or PDC. We also want to emphasize that few data are available for PDC compared to macrophages or conventional dendritic cells.

*3. When talking about the role of mTOR in pDCs, I think there is no need for a detailed introduction to mTOR – there are many recent authoratitive reviews on this subject. The authors should refer to one of these.  For example, a review by David Sabatini.*

We have quoted a review by David Sabatini (Ref# 8).

*4. The language of the review can be confusing at time. For example, the following sentence should probably be deleted for the sake of clarity: “After differentiation in the bone marrow, PDC are released into the bloodstream for homing to different lymphoid tissues. Thus, PDC isolated from blood of healthy donors or patients consist in PDC migrating to these tissues.”*

We have changed the beginning of this paragraph. We have deleted the second sentence since it corresponded to a repetition of the previous paragraph and we have modified a little bit the mentioned sentences.

*5. The authors should make an attempt to reference original work rather than other reviews. For example, “Localizations of PDC in other lymphoid organs, such as Peyer’s patches of the gut”. Instead of Li et al. (2011) ^1^, please cite the paper by Contractor et al. (2007) ^2^*.

Now, we have quoted the paper by Contractor et al. (2007) in addition to those by Li et al. (2011). Both papers are original works. We have also followed the recommendation to quote original works when references on PDC infiltrating tumors have been added.

# Peer review: Referee response for version 1

**DOI:** 10.5256/f1000research.12232.r21755

**Published:** 2017-05-11

**Authors:** Nathalie Bendriss-Vermare

**Affiliations:** 1 Team Caux (French National Institute of Health and Medical Research) INSERM U1052/CNRS 5286 (French National Center for Scientific Research), Cancer Research Centre of Lyon (CRCL), Lyon, France

This review provides a balanced and comprehensive overview of the latest discoveries about the role of metabolism in plasmacytoid dendritic cell innate functions. This is a very original topic based on recent data supporting the idea that the glycolytic pathway as well as lipid metabolism may modulate the production of type I IFN by pDC or may be involved in this function after TLR7/9 triggering. In addition, this review provides new clues on how these metabolic pathways may be harnessed in pathophysiological contexts where pDC play a detrimental role.

 This review has a comprehensive view of all relevant literature in the field and summarized very well the fundamental concepts, but there are some minor concerns that need to be addressed as below.

1. Abstract is clearly written, but the text could be reduced by omitting the section about the differences related to the origin of pDC.
2. Page 3: PRR stands for Pattern (but not Pathogen) Recognition Receptors
3. Page 3: I would suggest to add that i) human pDC are usually identified as CD4+ CD303+ CD123high and CD11cnegative (to highlight the difference with mouse pDC that are CD11clow), ii) irf7 is also a master genes that is shared between human and mouse pDC, iii) CD317 (known as BST2, PDCA1).
4. Page 5: the list of references related to the regulatory functions of pDC is too short. Some major articles should be added (Ochando 2006, Goubier 2008, Hadeiba 2008, Irla 2010, etc…).
5. Page 5 paragraph 2.4: when the authors discuss the role of pDC in diseases, they focus on autoimmune disorders, GVHD, atherosclerosis, and chronic viral infections but cancer was omitted. Yet, metabolic pathways are deeply altered in the context of cancer and I would assume that the innate functions of pDC would be modulated by the metabolic changes occurring in tumor microenvironment. This needs to be discussed.
6. Page 7: when the authors discuss the role of glycolysis on pDC functions upon TLR7 triggering, it is not clear whether this is a global regulation of the transcription or whether this is specific of genes related to type I IFN pathway.  
7. Page 10: it is not very interesting but not clear to me how the massive decrease of intracellular cholesterol content is connected to the activation of STING/cGAS pathway leading to spontaneous type I IFN production in macrophages. This should be clarified.
8. The figures nicely support and illustrate the main text of the review. Nevertheless, I would suggest to add a table summarizing the main effects of each metabolic pathway on pDC biology by comparing human versus mouse and TLR7 versus TLR9 stimulation.

I have read this submission. I believe that I have an appropriate level of expertise to confirm that it is of an acceptable scientific standard, however I have significant reservations, as outlined above.

# Response

**Published:** 2017-06-15

**Authors:** Philippe Saas

**Affiliations:** Department of Immunology and Cell Therapy, University of Bourgogne Franche-Comté, INSERM - EFS, France

We thank the reviewer for her constructive comments. We appreciate greatly these comments and we have tried to answer to them. Please find below a point by point response to the reviewer’s comments.

*1. Abstract is clearly written, but the text could be reduced by omitting the section about the differences related to the origin of pDC*.

We have modified the abstract accordingly.

*2. Page 3: PRR stands for Pattern (but not Pathogen) Recognition Receptors*

This is true. We have corrected this abbreviation.

*3. Page 3: I would suggest to add that i) human pDC are usually identified as CD4+ CD303+ CD123high and CD11cnegative (to highlight the difference with mouse pDC that are CD11clow), ii) irf7 is also a master genes that is shared between human and mouse pDC, iii) CD317 (known as BST2, PDCA1).*

We have added these features according to the reviewer’s suggestions.

*4. Page 5: the list of references related to the regulatory functions of pDC is too short. Some major articles should be added (Ochando 2006, Goubier 2008, Hadeiba 2008, Irla 2010, etc…).*

We have not expanded too much this part of the text. We have quoted the original work of Ochando 2006, Goubier 2008, and Hadeiba 2008, since it allows us to evoke briefly the role of PDC in transplantation and oral tolerance. However, we have not quoted the work of Irla (2010), since it deals with regulatory T cells and we focus our review on the innate immune functions of PDC.

*5. Page 5 paragraph 2.4: when the authors discuss the role of pDC in diseases, they focus on autoimmune disorders, GVHD, atherosclerosis, and chronic viral infections but cancer was omitted. Yet, metabolic pathways are deeply altered in the context of cancer and I would assume that the innate functions of pDC would be modulated by the metabolic changes occurring in tumor microenvironment. This needs to be discussed.*

This is a very interesting suggestion (please also refer to comment #1 of reviewer #1). Indeed, metabolism may play an important role in both tumor progression and antitumor immunity. Thus, a sentence has been added in the paragraph dealing with PDC ontogeny and localization, and different references describing PDC infiltrates in tumors have been quoted. We have quoted original works as advised by reviewers #3. Two small paragraphs have been added in the section called “2.4 Implications of PDC in diseases”. Again, references have been added in this section, including original works and reviews on metabolism and cancer. Moreover, we have also evoked quickly in different parts of the revised version that the tumor microenvironment may impact on PDC metabolism.

*6. Page 7: when the authors discuss the role of glycolysis on pDC functions upon TLR7 triggering, it is not clear whether this is a global regulation of the transcription or whether this is specific of genes related to type I IFN pathway.*

Glycolysis after TLR7 triggering on human PDC seems necessary for most of the PDC innate immune functions (e.g., type I IFN, costimulatory molecules). However, the authors concentrated most of their work on type I IFN pathway. In the text, we wrote “2-DG inhibits the increase of *IFNA*, *CD80* and CD86 mRNA induced by exposure to Flu ^4^ . This suggests that glycolysis induced by the TLR7 pathway regulates these genes at the transcriptional level.

*7. Page 10: it is not very interesting but not clear to me how the massive decrease of intracellular cholesterol content is connected to the activation of STING/cGAS pathway leading to spontaneous type I IFN production in macrophages. This should be clarified.*

This is true. The explanation provided in the first version of our manuscript was not clear. We have modified the sentence concerning this point.

*8. The figures nicely support and illustrate the main text of the review. Nevertheless, I would suggest to add a table summarizing the main effects of each metabolic pathway on pDC biology by comparing human versus mouse and TLR7 versus TLR9 stimulation.*

This is a very interesting suggestion. We have added a table summarizing the impact of pharmacological agents targeting the different metabolic pathways on TLR7/9 stimulation. Again, we focus on innate immune functions, including: type I IFN, IL-6 and TNF production as well as costimulatory molecule expression.

# Peer review: Referee response for version 1

**DOI:** 10.5256/f1000research.12232.r22503

**Published:** 2017-05-05

**Authors:** Vassili Soumelis, Francois Xavier Danlos

**Affiliations:** 1 Institut Curie, PSL Research University, Paris, France; 2 Institut Curie, Paris, France

This review by Saas et al. reports on an original question, rarely addressed in reviews: the role of metabolism on the innate functions of plasmacytoid dendritic cells. Authors have performed a very good job summarizing current knowledge in the field. They very extensively present the molecular and functional pathways involved in PDC biology, and the possible interactions with various metabolic pathways. This way represents the current state-of-the-art. A large number of references are cited, in the field of general PDC biology, including important founder papers, as well as in the field of metabolism, and the role of metabolic pathways in PDC.

 We only have a few comments on the manuscript:

1. The discussion of PDC implication in disease could be expanded. In particular, authors could discuss the role of PDC in the tumor microenvironment. It is known that metabolism plays an important role in tumor development and antitumor immunity. Can this also be through affecting PDCs? Is there a crosstalk between PDC biology, metabolism, and tumor progression? Several articles in the past few years have addressed the function of PDC in cancer and could serve as a basis for a discussion. Other diseases could also be discussed in more details, in particular autoimmunity. Indeed, the pathways involved in PDC activation in disease context may be slightly different from exogenous purified TLR ligands, due to the diversity of stimuli and the complexity of the inflammatory microenvironments.
2. Figure 2 is a bit too dense and confusing. We suggest to clarify it removing or reorganizing some of the information.
3. The article should also be edited for better English.

We have read this submission. We believe that we have an appropriate level of expertise to confirm that it is of an acceptable scientific standard.

# Response

**Published:** 2017-06-15

**Authors:** Philippe Saas

**Affiliations:** Department of Immunology and Cell Therapy, University of Bourgogne Franche-Comté, INSERM - EFS, France

We thank the reviewers for their constructive comments. We appreciate greatly these comments and we have tried to answer to them. Please find below a point by point response to the reviewers’ comments.

*1. The discussion of PDC implication in disease could be expanded. In particular, authors could discuss the role of PDC in the tumor microenvironment. It is known that metabolism plays an important role in tumor development and antitumor immunity. Can this also be through affecting PDCs? Is there a crosstalk between PDC biology, metabolism, and tumor progression? Several articles in the past few years have addressed the function of PDC in cancer and could serve as a basis for a discussion. Other diseases could also be discussed in more details, in particular autoimmunity. Indeed, the pathways involved in PDC activation in disease context may be slightly different from exogenous purified TLR ligands, due to the diversity of stimuli and the complexity of the inflammatory microenvironments.*

This is a very interesting suggestion (please also refer to comment #5 of reviewer #2). Indeed, metabolism may play an important role in both tumor progression and antitumor immunity. Thus, a sentence has been added in the paragraph dealing with PDC ontogeny and localization, and different references describing PDC infiltrates in tumors have been quoted. We have quoted original works as advised by reviewers #3. Two small paragraphs have been added in the section called “2.4 Implications of PDC in diseases”. Again, references have been added in this section, including original works and reviews on metabolism and cancer. Moreover, we have also evoked quickly in different parts of the revised version that the tumor microenvironment may impact on PDC metabolism. However, to the best of our knowledge, no specific data are available today. We do not want to speculate too much on the impact of tumor microenvironment on PDC functions. We have made no further comments on autoimmunity and the diversity of stimuli.

*2. Figure 2 is a bit too dense and confusing. We suggest to clarify it removing or reorganizing some of the information.*

We agree with this comment. We have simplified both panels A and B of Figure 2. In addition, we have modified a little bit the figure legend.
